# Assessment of Oral Health Status and Treatment Needs of Institutionalized Children With Special Needs in Poonamallee, Chennai: A Cross-Sectional Study

**DOI:** 10.7759/cureus.48139

**Published:** 2023-11-02

**Authors:** Indumathy Pandiyan, Meignana Arumugham I, Srisakthi D, Jayashri Prabakar

**Affiliations:** 1 Public Health Dentistry, Saveetha Dental College and Hospitals, Saveetha Institute of Medical and Technical Sciences, Saveetha University, Chennai, IND

**Keywords:** physically challenged, intellectually disabled, oral health assessment, disability and inclusion, children with special needs

## Abstract

Background

Dental caries represents a pervasive chronic pediatric ailment that significantly hinders normal patterns of nutrition intake, speech articulation, and daily activities. Notably, children with special needs emerge as a particularly susceptible demographic concerning dental afflictions, specifically in the context of dental caries and periodontal diseases. The objective of this study was to assess the oral health status of children with special needs in Poonamallee, Chennai, India.

Methodology

This cross-sectional survey involved a total of 1,114 children with special needs, classified into two groups, namely, intellectually disabled and physically challenged. The assessment of various oral health parameters in the study population was performed using the World Health Organization’s Oral Health Assessment Form for Children 2013. For quantitative variables, mean and standard deviations were considered. The significance of the difference between quantitative variables was tested using an independent t-test. Statistical significance was set at p-values ≤0.05.

Results

Of the 1,114 children, 552 were females and 562 were males. Overall dental trauma was noted in 21%. The average decayed missing filled teeth (DMFT) value was 0.74, with intellectually disabled children exhibiting a higher mean compared to the physically challenged group with a mean value of 0.72. Additionally, when assessing gingival conditions, children with intellectual disabilities displayed more unfavorable outcomes compared to their physically challenged counterparts. A need for dental intervention was observed in 97.3% of the pediatric population. A statistically significant difference was found for missing teeth (M) in the DMFT index between the intellectually disabled and physically challenged groups.

Conclusions

The results of this study emphasize the less-than-ideal oral health condition among children with special needs, highlighting the urgent necessity for the creation of a comprehensive dental healthcare program tailored to their specific needs.

## Introduction

A child who, due to various factors, is unable to fully utilize their physical, mental, and social abilities is described as having a disability, According to the definition of the American Health Association [1]. More than 26.8 million people in India have a disability of one kind or another, according to the 2011 census. Overall, 15 million of the 26.8 million disabled people in the country are men and 11.8 million are women. According to the census, physically disabled people account for 20.3%, followed by hearing-impaired people (18.9%), and visually impaired people (18.9%). The intellectually disabled category accounts for around 5.6% of the impaired population [2].

Oral health has a major biological, psychological, and social impact on one’s aesthetic and communicative abilities [3]. Unfortunately, one of the top unmet health requirements of people with disabilities is oral care. Children with disabilities may exhibit more pronounced oral pathologies, which can be attributed to both their underlying disabilities and a combination of additional medical, financial, or social factors. Moreover, it is possible that the caregivers of these children encounter considerable difficulties in maintaining effective daily oral hygiene practices, potentially exacerbated by factors such as the cariogenic impact of medications containing high sugar content and persistent tooth-grinding behaviors coupled with self-mutilation tendencies [4]. Researchers worldwide have collectively established a higher incidence and increased severity of oral disorders among individuals with disabilities [3,5-7], with a particular focus on the Indian context [8-12].

All children are different in their own way. Oral health issues are common among children with special healthcare requirements, making it difficult for parents and other caregivers to provide oral hygiene care. Nevertheless, it is important for us to understand as medical professionals that caring for a child with unique needs can inspire one to become a truly exceptional individual. Considering the information mentioned earlier, we employed the World Health Organization (WHO) Oral Health Assessment Form 2013 [13] to create a comprehensive oral health survey. This survey aimed to provide a thorough understanding of the oral health condition of children between 3 and 14 years old who attended various special schools in the Poonamallee region of Chennai, specifically those with special needs.

## Materials and methods

A cross-sectional descriptive survey was conducted to evaluate the oral health status and treatment requirements of two primary disability groups, namely, individuals with intellectual disabilities and those with physical challenges. The sample size for this study was determined based on data obtained from a pilot study involving 50 subjects within a similar age group conducted at the Outpatient Department of Public Health Dentistry. The prevalence of dental caries was considered when calculating the sample size, which was derived to be 1,114. The following formula was used for calculating the sample size: sample size (n) = z^2^pq/d^2^, where n = desired sample size, z = level of significance at 95% CI (1.96), p = proportion of the study population, q = 1-p, and d = degree of accuracy desired, usually set at 0.05.

Ethical clearance and informed consent

Before conducting clinical assessments on the children, informed consent was obtained from their parents or guardians, and group consent was also secured from the relevant school authorities. Study approval was granted by the Ethical Research Committee of Saveetha University, Chennai, ensuring adherence to ethical guidelines. Among the eight institutions initially shortlisted from Chennai’s Poonamallee district for participation, five granted permission to participate in the study.

Training and calibration of examiners

To ensure the reliability and consistency of the data collection process, two researchers, supervised by a principal investigator, were involved in data collection. The pilot study was utilized to train and calibrate the examiners, and Cohen’s kappa coefficient for assessing dental caries demonstrated a high level of interexaminer reliability and agreement, with a coefficient value of 0.82.

Inclusion and exclusion criteria

The study included children between the ages of three and 14 who were present on the day of the examination. Exclusion criteria encompassed children who were unwilling to participate or were experiencing health issues. Data collection occurred over two months.

Data recording

Participants participated in oral examinations while seated in regular chairs in their individual schools. The American Dental Association’s recommendation for Type III clinical examination using a mouth mirror and probe under suitable lighting was followed. A mouth mirror and Community Periodontal Index (CPI) probe were used for the examination. Teachers’ and the caregivers’ assistance was used to communicate with the children.

The WHO Oral Health Assessment Form 2013 (Appendices) [13] for children was used to document their oral health status. This method of data collection contained general information about the study subjects, such as name, date of birth, age, and gender. A clinical evaluation included noting the child’s dentition status for caries and any consequences, such as missing or filled teeth because of caries. The CPI probe was used to record gingival bleeding to determine the gingival condition. Dental trauma using Ellis and Davey’s classification (1960) and dental fluorosis using Deans’s Fluorosis Index, modified criteria (1942) were also evaluated to assess presence and severity.

If a tooth did not erupt after six months from the anticipated eruption date, it was designated as missing. If a tooth was still in the arch six months after it was supposed to exfoliate, it was retained. According to the type of treatment needed for a particular child, the urgency of interventions and treatment needs were also determined.

Statistical analysis

Data analysis was done using SPSS version 17 (SPSS Inc., Chicago, IL, USA). As the sample size was large, the Kolmogorov-Smirnov normality test was performed, and it was found that the data were following normal distribution. Qualitative variables were calculated using frequencies and percentages. Mean and standard deviations were calculated for quantitative variables. The independent t-test was used to test the significance of differences between quantitative variables. The statistical significance level threshold was established at p-values ≤0.05.

## Results

**Figure 1:**
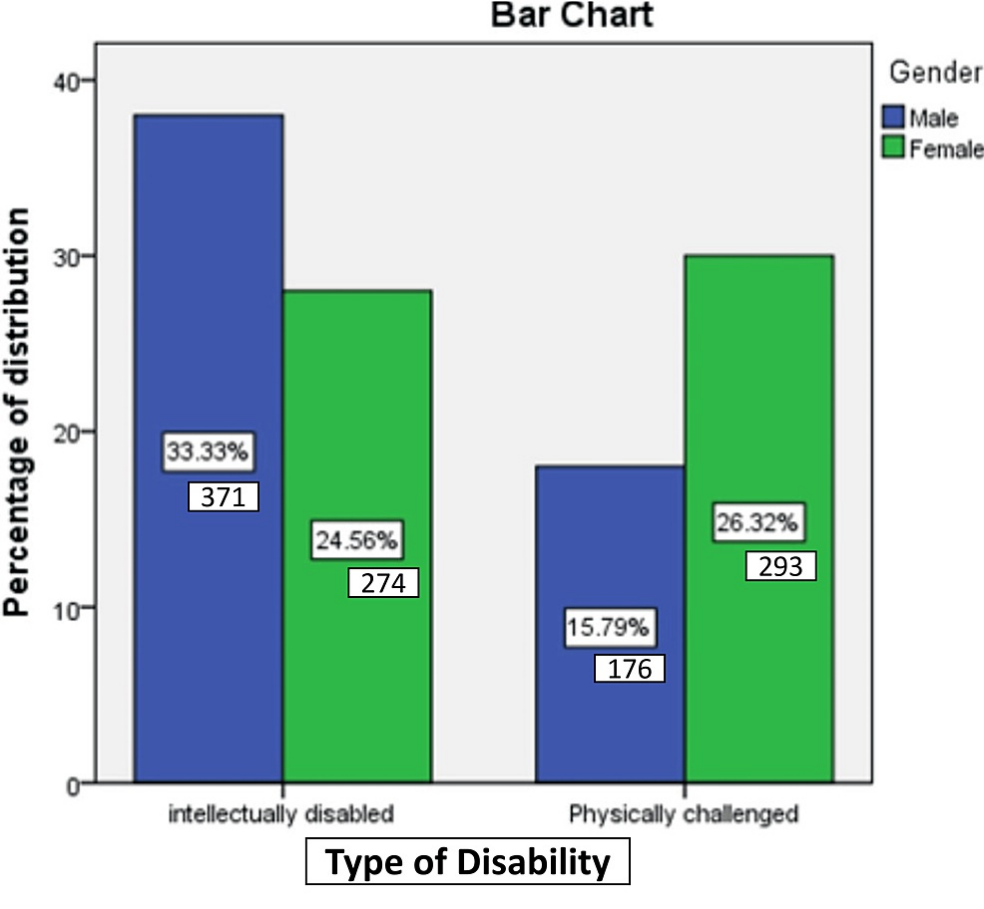
Distribution of study participants according to the type of disability and gender.

The sample for this study comprised a total of 1,114 children, with males representing the majority at 49.12% of the total population (Figure 1).

Mean and standard deviation values for decayed, missing, filled teeth (DMFT) were computed separately for both groups and are presented in Table 1. Notably, decayed teeth in intellectually disabled children constituted the largest portion when compared with the physically challenged group, whereas missing teeth were more prevalent among the physically challenged children. Upon conducting an independent t-test for DMFT in both groups (Table 2), it was observed that children with intellectual disabilities had a higher mean DMFT when compared to the physically challenged group.

**Table 1:** Comparison of mean DMFT values among the groups. *: Statistically significant difference between groups. DMFT = decayed, missing, filled teeth

Disability group	Decayed teeth (mean ± SD)	Missing teeth (mean ± SD)	Filled teeth
Intellectually disabled	0.72 ± 1.19	0.15 ± 0.12	0
Physically challenged	0.60 ± 1.14	0.33 ± 0.83	0
P-value	0.5	0.00*	0

**Table 2:** Comparison of mean DMFT using independent t-test. *: Statistically no difference between groups. DMFT = decayed, missing, filled teeth

Disability group	DMFT (mean ± SD)	Independent t-test	P-value
Intellectually disabled	0.74 ± 1.20	1,579.50	0.9*
Physically challenged	0.72 ± 1.21		

In the primary dentition, mean and standard deviation values for decayed, extracted, and filled index (def-index) were also calculated separately for both groups and are presented in Table 3. An independent t-test for def-index (Table 4) revealed that physically debilitated children had comparatively less significant mean def-index values compared to those with intellectual disabilities (p < 0.3).

**Table 3:** Comparison of mean def-index values among intellectually disabled and physically challenged groups. *: Statistically no difference between groups. def = decayed, extracted, and filled

Disability group	Decayed teeth (mean ± SD)	Extracted due to caries (mean ± SD)	Filled teeth
Intellectually disabled	0.51 ± 1.30	0	0
Physically challenged	0.60 ± 1.14	0	0
P-value	0.2*	0	0

**Table 4:** Comparison of mean def-index values using independent t-test. *: Statistically no difference between groups. def = decayed, extracted, and filled

Disability group	Mean def (Mean ±SD)	Independent t	P value
Intellectually disabled	0.51±1.30	1458.00	0.3*
Physically Challenged	0.33±0.83		

The study revealed an overall dental trauma prevalence of 21%, with a higher incidence observed among physically disabled children in comparison to the other group. The mean values of gingival bleeding and dental trauma among both groups are indicated in Table 5. Furthermore, 22% of the children displayed signs of gingival bleeding, with no statistically significant difference observed between the groups. However, it is worth noting that intellectually disabled children had slightly elevated scores for gingival bleeding.

**Table 5:** Comparison of mean gingival bleeding and dental trauma values among the intellectually and physically disabled groups.

Disability group	Gingival bleeding (mean ± SD)	Dental trauma (mean ± SD)
Intellectually disabled	0.24 ± 0.43	0.42 ± 0.94
Physically challenged	0.18 ± 0.39	0.66 ± 1.20

**Figure 2:**
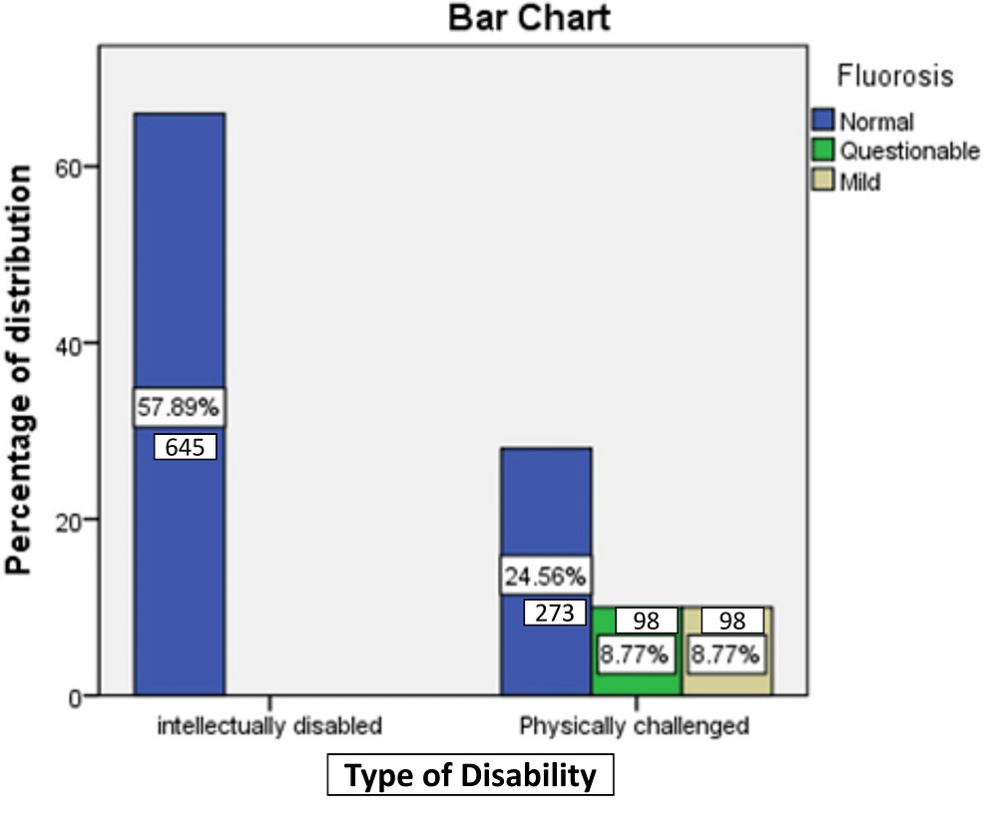
Distribution of participants according to the presence or absence of enamel fluorosis. Questionable fluorosis and mild fluorosis were observed in 8.7% of cases among physically challenged individuals, but there were no occurrences of questionable and mild fluorosis in intellectually disabled individuals.

Enamel fluorosis of varying severity levels is depicted in Figure 2. Among physically challenged individuals, questionable fluorosis and mild fluorosis were present in 8.7% of cases, while there were no instances of fluorosis among intellectually disabled individuals. Only 33 out of the 1,114 children did not require any form of dental treatment. Approximately 71% of children needed prompt dental treatment, including scaling, while 26.3% required preventive or routine treatment. None of the children fell into the category of requiring immediate treatment due to pain or infection of dental/oral origin. Significantly, there was no statistically noteworthy distinction between the disability groups concerning the type of intervention needed (p = 0.2), as presented in Table 6.

**Table 6:** Distribution of participants according to treatment needs.

Disability group	No treatment required	Preventive or routine treatment needed	Prompt treatment needed	Immediate treatment needed
Intellectually disabled	33	145	423	0
Physically disabled	0	145	367	0
Total (%)	33 (2.6%)	290 (26.3%)	790 (71%)	0
Chi-square value	3.01			
P-value	0.22			

## Discussion

Overall health and well-being are correlated with oral health. Unfortunately, many children’s dental ailments go undiagnosed because of their circumstances and a lack of knowledge about oral health, which results in a significant unmet need for dental care later in life. A cross-sectional descriptive study was conducted in several special needs schools in Chennai, India. In total, 1,114 children, 562 boys and 552 girls, were evaluated across six institutes for a variety of oral disorders and treatment requirements.

Dental caries has emerged as a growing oral health concern among Indian children. According to a National Oral Health Survey conducted during 2003-2004, the mean DMF values were reported as 2, 1.8, and 2.3, respectively, with caries prevalence rates of 51.9%, 53.8%, and 63.1% at ages five, 12, and 15, respectively [14]. Notably, the mean DMF among 12-year-old children in India ranged from 1.2 to 2.6, as documented in the 2003 WHO Oral Health Report [15].

In this study, the mean DMF value was recorded as 1.46, aligning reasonably well with the findings from the nationwide surveys mentioned earlier. This suggests that the severity of caries might be relatively higher, even though only a limited number of children were affected with dental caries compared to the general child population. Another contributing factor could be previous tooth extractions or periodontal issues, which is particularly noteworthy among individuals with severe intellectual disabilities who are living in institutional settings [16].

Notably, in various studies conducted in India, the mean DMF values in children with intellectual disabilities were lower [10,11]. Similarly, when compared to research involving special needs children in Bhopal [17] and Delhi [18], children in Udaipur and Delhi who were visually and hearing impaired exhibited somewhat lower mean DMF values [9,19].

Several studies [8,20,21] and a systematic review [22] have highlighted deficient oral hygiene, especially among children with cognitive impairments, in comparison to the general population.

In urban areas, 96.4% of individuals were observed to use toothbrushes, in contrast to 91.8% in rural regions. Furthermore, 100% of urban residents practiced daily toothbrushing, while 99.8% in rural areas did the same. These findings are consistent with those reported in previous studies [23,24].

When comparing treatment needs between rural and urban populations, it became apparent that the rural population had higher treatment requirements. These needs included oral prophylaxis, one-surface fillings, crowns, extractions, and pulp therapy. This discrepancy can be attributed to factors such as limited knowledge and awareness among parents and caregivers, the lower socioeconomic status of parents and guardians, a lower priority placed on oral health, restricted access to early and regular oral health check-ups, and the financial burden associated with dental treatment [25,26].

In this study, the overall prevalence of gingival bleeding was 59.09%. It is worth highlighting that individuals with intellectual disabilities displayed the least favorable oral hygiene, with 69.9% experiencing gingival bleeding. Conversely, those with physical impairments demonstrated better gingival health in comparison to the intellectually disabled group. These results are consistent with findings reported in a study conducted by Shukla et al. [19].

Various factors can be attributed to these findings, such as the limited intellectual abilities that may hinder the adoption of effective oral hygiene practices. While individuals with visual impairments may comprehend dental hygiene instructions and possess typical kinematic abilities, their ability to visually identify and remove plaque can be compromised. Consequently, the pivotal determinant of oral health status appears to be the nature of the disability and how it affects the ability to maintain adequate or optimal oral hygiene practices [20].

In response to these challenges, several specially designed manual toothbrushes have been developed to facilitate comprehensive cleaning of tooth surfaces with a single stroke. One notable example is the triple-headed brush, which is recommended for individuals with limited manual dexterity [27].

In this study, a significant finding revealed a heightened prevalence of dental trauma, with a specific impact on the maxillary incisors, among female children with physical challenges. This can be attributed to their limited mobility, rendering them more vulnerable to accidents and falls resulting from walking difficulties. This observation underscores the significant unmet demand for dental care within both disability groups.

It is imperative to recognize specific constraints inherent to this study, which encompass the utilization of a convenience sampling methodology. Additionally, the investigation did not include an assessment of the extent of disabilities, proficiency in motor skills, or the multifaceted social and behavioral determinants that may be interlinked with oral health dynamics within the studied population.

## Conclusions

The study outcomes reveal that the oral health condition of children with special needs is markedly subpar, marked by a limited rate of treatment utilization and a significant volume of unfulfilled requirements. Specifically, children with intellectual disabilities exhibited a significantly higher mean DMFT, and this group was also more susceptible to poor gingival conditions. In contrast, dental trauma was more frequently observed among physically challenged children.

## Figures and Tables

**Figure 3:**
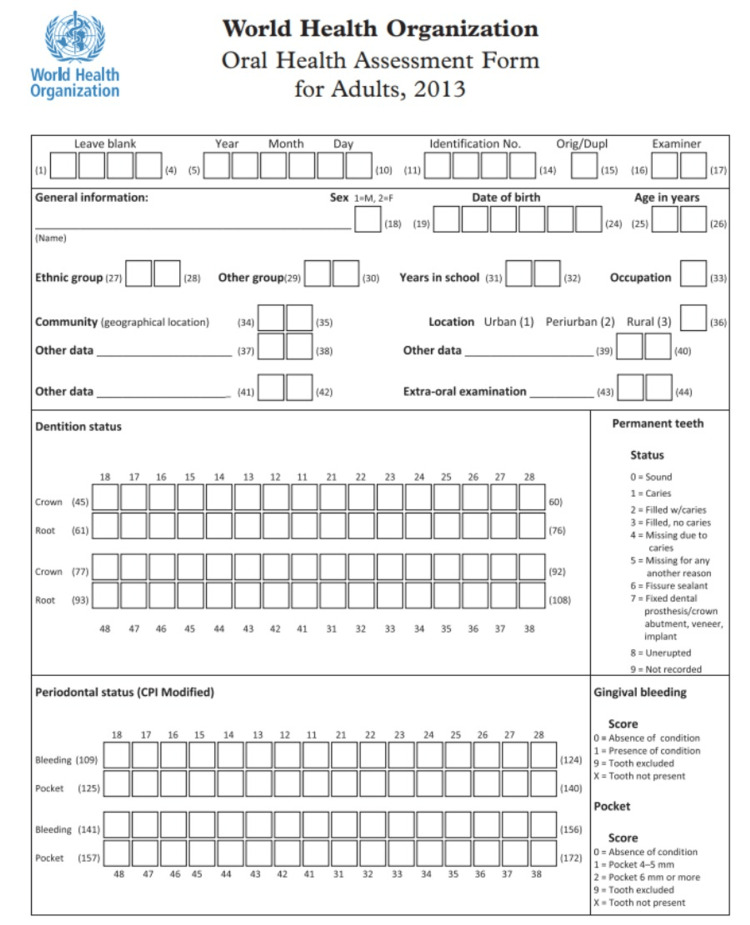
World Health Organization’s Oral Health Assessment Form (2013): part 1.

**Figure 4:**
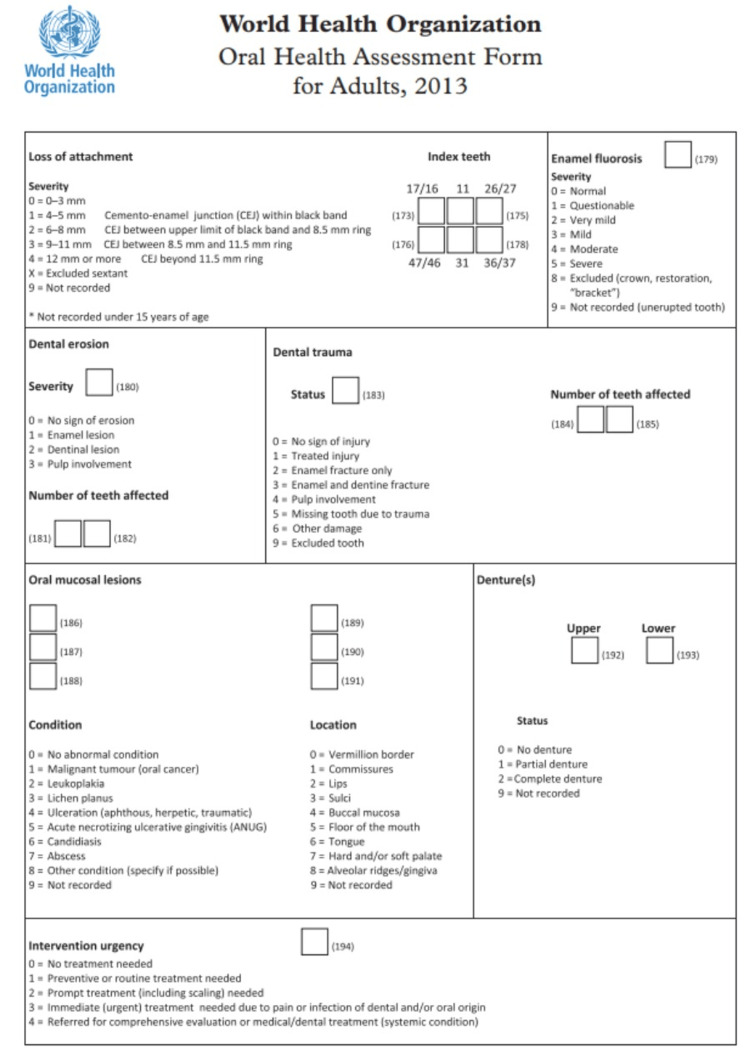
World Health Organization’s Oral Health Assessment Form (2013): part 2.
